# Apigenin facilitates apoptosis of acute lymphoblastic leukemia cells via AMP-activated protein kinase-mediated ferroptosis

**DOI:** 10.32604/or.2024.049757

**Published:** 2025-01-16

**Authors:** CANCAN HE, TINGTING ZHANG, WEI XIONG, SHENGYU WANG, XIN SUN

**Affiliations:** 1 Department of Pediatrics, Affiliated Hospital of Zunyi Medical University, Zunyi, 563003, China; 2 Department of Pediatrics, Guizhou Children’s Hospital, Zunyi, 563003, China; 3 Department of Radiology, Affiliated Hospital of Zunyi Medical University, Zunyi, 563003, China; 4 Department of Cardiovascular Surgery, Affiliated Hospital of Zunyi Medical University, Zunyi, 563003, China; 5 Key Laboratory of Infectious Disease & Biosafety, College of Preclinical Medicine, Zunyi Medical University, Zunyi, 563003, China; 6 Department of Microbiology, College of Preclinical Medicine, Zunyi Medical University, Zunyi, 563003, China

**Keywords:** Acute lymphoblastic leukemia (ALL), Apigenin, Apoptosis, AMP-activated protein kinase (AMPK), Ferroptosis

## Abstract

**Background:**

The outcomes of pediatric patients with acute lymphoblastic leukemia (ALL) remain far less than favorable. While apigenin is an anti-cancer agent, studies on the mechanism by which it regulates ALL cell cycle progression are inadequate. Ferroptosis and AMP-activated protein kinase (AMPK) signaling are important processes for ALL patients. However, it remains unclear whether apigenin works by affecting AMPK and apoptosis.

**Materials and Methods:**

SUP-B15 and T-cell Jurkat ALL cells were treated with apigenin, and cell viability and apoptosis were measured using 3-(4,5-dimethylthiazol-2-yl)-2,5-diphenyltetrazolium bromide (MTT) and terminal deoxynucleotidyl transferase dUTP nick end labeling (TUNEL) assays, respectively. The thiobarbituric acid-reactive substances (TBARS) assay was used to evaluate lipid peroxidation. Intracellular Fe^2+^ levels were measured using a commercial kit. Corresponding proteins were detected by western blotting.

**Results:**

Results showed that apigenin reduced cell viability and the levels of Ki67 and proliferating cell nuclear antigen (PCNA) expression in a concentration-dependent manner in both types of ALL cells. Apigenin also exerted anti-apoptotic effects on SUP-B15 and Jurkat cells. Apigenin activated AMP-activated protein kinase (AMPK) signaling and induced ferroptosis, and those effects were attenuated by inhibition of AMPK. Eventually, the reduced cell proliferation and increased cell apoptosis caused by apigenin in ALL cells were partly abolished by AMPK inhibition.

**Conclusion:**

In summary, apigenin exerted anti-leukemia activity in ALL cells, and that effect was partially achieved by activation of AMPK signaling. Our findings suggest apigenin as a potential drug for treatment of ALL.

## Introduction

Acute lymphoblastic leukemia (ALL) is a hematological malignant disease characterized by the rapid and excessive proliferation of lymphoblasts originating from B or T cells [1,2]. The clinical manifestations of ALL include reduced hematopoiesis, anemia, neutropenia, thrombocytopenia, and hepatosplenomegaly. T-cell ALL in children is proven to be more dangerous than that of B-cell ALL [3]. In recent decades, effective therapeutic strategies have evolved, and contemporary childhood ALL studies have indicated that the 5-year overall survival rate now exceeds 82.6% and the event-free survival rate exceeds 75.2% [4–6]. However, pediatric patients often relapse, and the outcomes for pediatric patients with recurrence or resistance remain far less favorable [7,8]. As a result, attempts are currently being made to find better treatments for ALL.

**Figure 1:**
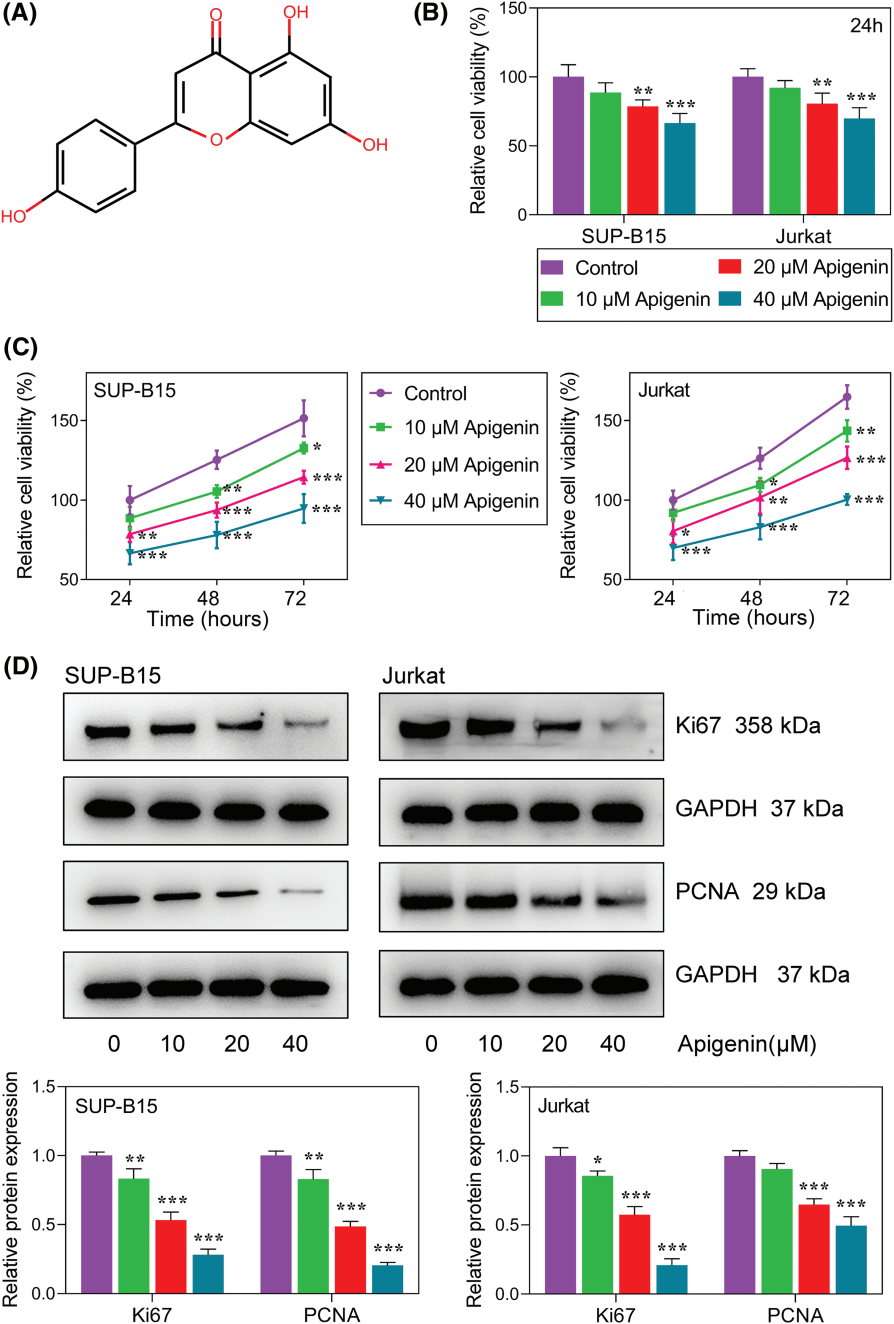
Apigenin inhibited ALL cell proliferation. (A) The chemical structure of apigenin. (B) The viability of SUP-B15 and Jurkat cells at 24 h after treatment with graded concentrations of apigenin was detected using the MTT assay. (C) The viability of SUP-B15 and Jurkat cells at different time points following treatment with graded concentrations of apigenin was detected using the MTT assay. (D) The levels of Ki67 and PCNA expression were detected by western blotting. *p < 0.05, **p < 0.01, ***p < 0.001.

After considering the benefits of multiple therapeutic effects offered by natural compounds used in traditional medicine, scholars have focused much of their attention on the anti-tumor effects of natural compounds and subsequently attempted to understand their mechanisms of action. Accordingly, it has been pointed out that apigenin (4′,5,7-trihydroxyflavone; Fig. 1A) is commonly present in onions, oranges, parsley, grapefruit, and chamomile, exhibits a broad spectrum of activities attributable to its regulatory effects on multiple intracellular targets [9,10]. Studies demonstrated that apigenin can induce cell apoptosis and is a potential inhibitor of cell growth in cancers, including colorectal cancer, lung cancer, cervical cancer, and hepatocellular carcinoma cells, being one promising drug for cancer therapeutic [11–14]. The anti-leukemia activity of apigenin has been proven in chronic myeloid leukemia cells and monocytic leukemia cells. Nonetheless, it remains unclear whether apigenin can induce apoptosis in ALL cells and exert anti-leukemia activity in ALL.

Ferroptosis and AMP-activated protein kinase (AMPK) are both essential for regulating cell death. Apigenin promotes ferroptosis in human endometrial cancer [15] and intestinal ischemia reperfusion injuries [16]. However, it has not been reported whether apigenin exerts its effects via ferroptosis or the AMPK signaling pathway in ALL.

To determine the molecular mechanism of apigenin in ALL, we investigated the effects of apigenin on ALL cell progression in human B-cell ALL cells (SUP-B15) and T-cell ALL cells (Jurkat). Furthermore, we also explored the effect of apigenin on ferroptosis and the AMPK signaling pathway.

## Materials and Methods

### Cell culture and treatment

SUP-B15 and Jurkat ALL cells were purchased from the American Type Culture Collection (ATCC; Manassas, VA, USA).

SUP-B15 cells were cultured in Iscove’s modified Dulbecco’s medium (IMDM; Cat: 51471C, Sigma-Aldrich Inc., St. Louis, MO, USA) supplemented with 20% fetal bovine serum (FBS; Cat:A5669701, Life Technologies, Carlsbad, CA, USA). Jurkat cells were cultured in Roswell Park Memorial Institute medium (RPMI-1640, Cat:12633012, Thermo Fisher Scientific, Waltham, MA, USA) supplemented with 10% FBS. All cells were cultured at 37°C in a 5% CO_2_ atmosphere.

SUP-B15 and Jurkat cells were treated with graded apigenin (Cat:SMB00702, Sigma-Aldrich; 0, 10, 20, and 40 µM) for 24 h. They were also pre-treated with 10 μM compound C (an AMP-activated protein kinase inhibitor; Cat:P5499, Sigma-Aldrich) or 0.1 μM Fer-1 (a ferroptosis inhibitor; Cat:SML0583, Sigma-Aldrich) for 24 h for the purpose of exploring regulatory mechanisms. All drugs were dissolved in dimethyl sulfoxide.

### 3-(4,5-dimethylthiazol-2-yl)-2,5-diphenyltetrazolium bromide (MTT) assay

About 2 × 10^3^ SUP-B15 and Jurkat cells were inoculated into 96-well plates and incubated with graded apigenin for 24, 48 and 72 h, respectively. Next, the cells were treated with 5 mg/mL MTT solution (Cat:C0009M, Beyotime Biotechnology, Shanghai, China) at 37°C for 4 h, and subsequently treated with 100 μL of dimethylsulfoxide (DMSO, Millipore Sigma, Burlington, MA, USA). The absorbance of each sample at 570 nm was detected using a microplate reader (Thermo Fisher Multiskan FC, Waltham, MA, USA).

### Western blotting

Total cellular protein was extracted and evaluated by cell lysis buffer containing a protease/phosphatase inhibitor cocktail (Cat:#5872, Cell Signaling Technology, Danvers, MA, USA) and Pierce BCA Protein Assay Kit (Cat:23227, Thermo Fisher Scientific). Standard sodium dodecyl sulfate-polyacrylamide gel electrophoresis (SDS-PAGE) methods were used in this study. Briefly, the gels were transferred onto polyvinylidene fluoride (PVDF, Cat:22860, Thermo Fisher Scientific) membranes, blocked with 5% skimmed milk, (Cat:37530, Thermo Fisher Scientific) and incubated with primary antibodies (1:1000) at 4°C overnight; after which, they were washed and subsequently incubated with horseradish peroxidase (HRP) (Cat: ab6721, Abcam, 1:10000)-conjugated secondary antibody at 25°C for 2 h. The immunoreactive protein bands were visualized using an enhanced chemiluminescence kit (Cat: WBAVDCH01, Millipore, Billerica, MA, USA). All antibodies were obtained from Abcam (Cambridge, MA, USA), and GAPDH served as an internal control.

### Apoptosis analysis

The terminal deoxynucleotidyl transferase dUTP nick end labeling (TUNEL) assay was employed for cell apoptosis evaluation. In brief, cells were fixed with 4% paraformaldehyde and permeabilized with 0.3% Triton X-100 for 5 min at 25°C. The cells were then incubated with TUNEL reagent (Cat: C1088, Beyotime Biotechnology) for 60 min in the dark. After washing, images were captured under one fluorescence microscope (Cat: FV4000MPE, Olympus Corporation, Tokyo, Japan).

### Thiobarbituric acid-reactive substances (TBARS) assay

The TBARS assay was used to detect lipid peroxidation [17]. Briefly, the supernatants of cell lysates were collected and treated with 0.1% thiobarbituric acid solution at 95°C for 30 min. The absorbance of each supernatant at 532 nm was recorded under one microplate reader (Cat:1410101, Thermo Fisher Multiskan FC).

### Iron level assay

Intracellular Fe^2+^ levels were measured with an Iron Assay Kit (Cat: ab83366, Abcam, Cambridge, UK) referring to the protocol [18].

### Reactive oxygen species testing

The level of reactive oxygen species (ROS) was detected with a commercial ROS Assay Kit (Cat: S0035M, Beyotime Biotechnology).

### Statistical analysis

All data and the graphs were analyzed and drawn using GraphPad Prism software (version 8.0; Graphpad Prism software, La Jolla, CA, USA). Data are presented as a mean value ± standard deviation (SD). One-way ANOVA followed by Tukey’s *post hoc* test was employed for statistical analysis. All experiments were tripled. *p*-value < 0.05 was considered to be statistically significant.

## Results

### Apigenin inhibited ALL cell proliferation

The chemical structure of apigenin is shown in Fig. 1A. We first examined the cytotoxicity of apigenin in cells. As shown in Fig. 1B, their viability was markedly reduced (*p* < 0.01, *p* < 0.001) when treated with graded apigenin, demonstrating that apigenin reduced the viability of ALL cells. Next, we examined its role in regulating ALL cell proliferation. As shown in Fig. 1C, increasing concentrations of apigenin reduced the proliferation of SUP-B15 and Jurkat ALL cells in a concentration-dependent manner (*p* < 0.05, *p* < 0.01, *p* < 0.001). Furthermore, we also detected the expression levels of classical proliferation-related proteins, including Ki67 and proliferating cell nuclear antigen (PCNA) by western blotting. As expected, treatment with apigenin remarkably reduced the levels of Ki67 and PCNA protein expression in a concentration-dependent manner in both SUP-B15 and Jurkat cells (Fig. 1D, *p* < 0.05, *p* < 0.01, *p* < 0.001).

### Apigenin induced ALL cell apoptosis

**Figure 2:**
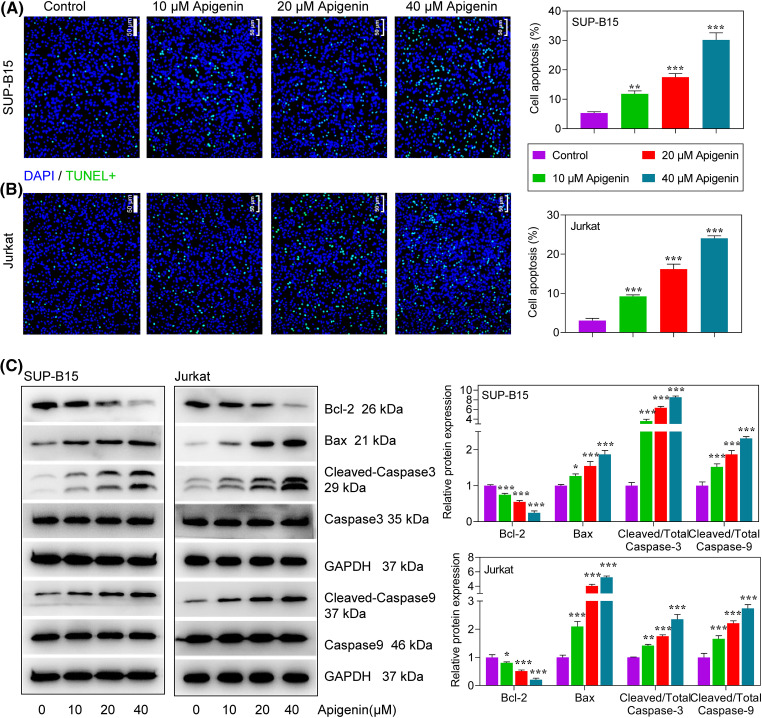
Apigenin induced apoptosis in ALL cells. (A and B) TUNEL assays were conducted to detect the apoptosis of SUP-B15 and Jurkat cells after treatments. Scale bar = 50 μm. (C) The expression of apoptosis-related proteins at 24 h after treatment was determined by western blotting. *p < 0.05, **p < 0.01, ***p < 0.001.

Subsequently, we examined how apigenin affected the apoptosis of SUP-B15 and Jurkat cells. The percentages of TUNEL-positive cells subsequently increased with the apigenin concentrations added. Furthermore, an increasing trend was observed in both SUP-B15 and Jurkat cells (Figs. 2A and 2B), suggesting a pro-apoptotic role of apigenin on ALL cells. To verify this finding, we examined the expression levels of pro- and anti-apoptotic proteins in treatments. We found that apigenin significantly reduced Bcl-2 protein expression and increased that of Bax, cleaved caspase-3, and cleaved caspase-9 in a concentration-dependent manner (*p* < 0.05, *p* < 0.01, *p* < 0.001, Fig. 2C).

### Apigenin activated AMPK signaling and ferroptosis in leukemia cells

**Figure 3:**
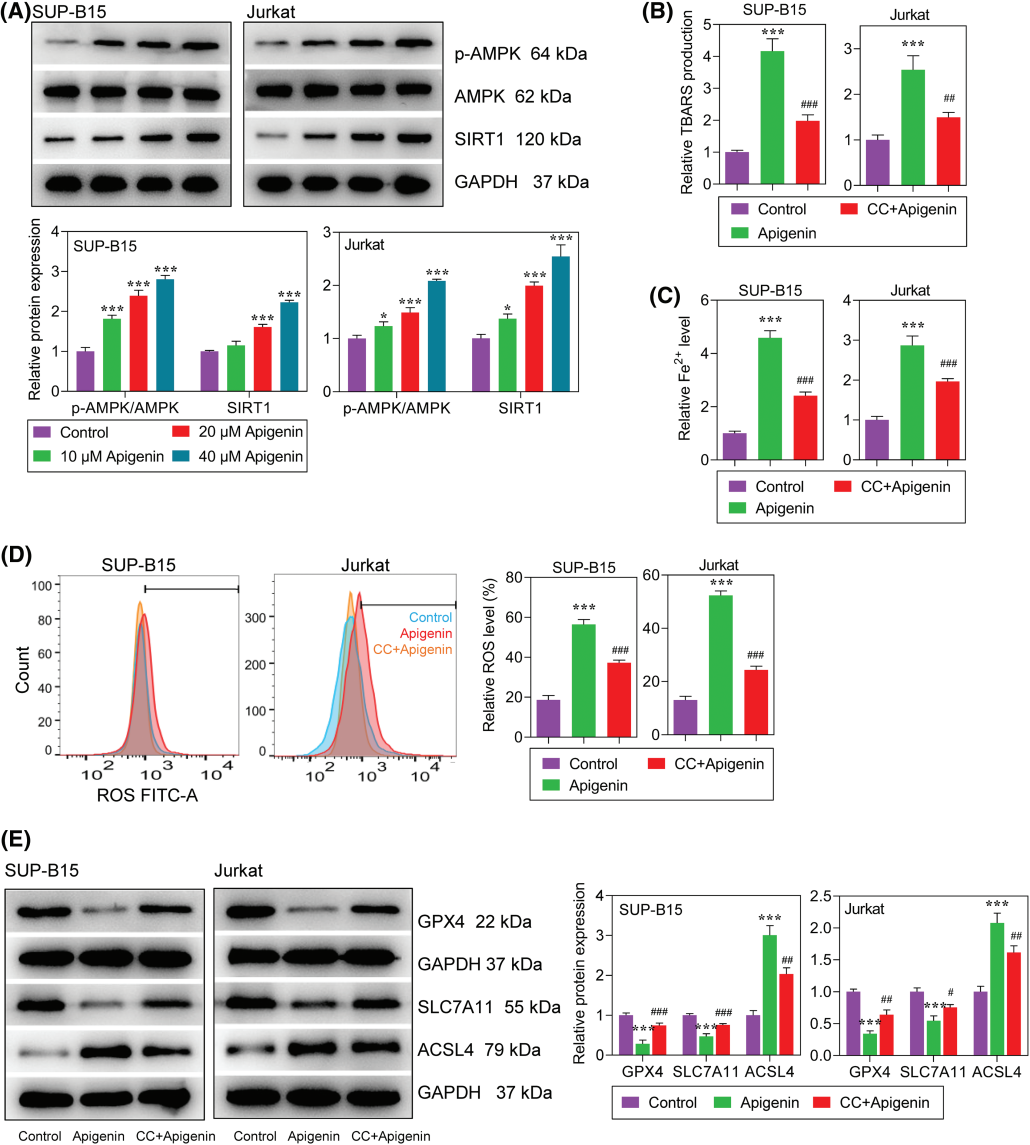
Apigenin activated AMPK signaling and ferroptosis in leukemia cells. (A) The levels of phosphorylated (p)-AMPK and SIRT1 expression at 24 h after treatment were detected by western blotting. (B) Lipid peroxidation at 24 h after treatment was determined by the TBARS assay. (C) Intracellular Fe^2+^ levels at 24 h after treatment were measured using an Iron Assay Kit. (D) Reactive oxygen species (ROS) at 24 h after treatment were detected by flow cytometry. (E) The levels of GPX4, SLC7A11, and ACSL4 protein expression at 24 h after treatment were detected by western blotting. *p < 0.05, ***p < 0.001 *vs*. Control; ^#^*p* < 0.05, ^##^*p* < 0.01, ^###^*p* < 0.001 *vs*. apigenin.

The molecular mechanism of apigenin in regulating ALL cells through AMPK signaling and ferroptosis was determined. The levels of phosphorylated (p)-AMPK and silent information regulator 1 (SIRT1) expression in SUP-B15 and Jurkat cells were significantly increased in a concentration-dependent manner after treatment with apigenin (Fig. 3A), indicating that apigenin could activate AMPK signaling in ALL cells. Next, to better explore the regulatory mechanism of apigenin, a 40 μM concentration of apigenin was selected for use in subsequent experiments that were conducted with or without pre-treatment with CC, an AMPK inhibitor. Our results showed that apigenin greatly improved the TBARS products in SUP-B15 and Jurkat cells, indicating that lipid peroxidation was elevated after treatment; however, this elevation was attenuated by additional treatment with CC (Fig. 3B). Meanwhile, we also found that apigenin significantly elevated Fe^2+^ and ROS levels, which were significantly decreased by CC+Apigenin (*p* < 0.001, Figs. 3C and 3D). In addition, apigenin significantly decreased the levels of glutathione peroxidase 4 (GPX4) and solute carrier family 7-member 11 (SLC7A11), increased that of acyl-CoA synthetase long-chain family member 4 (ACSL4) expression in ALL cells, and those regulation trends were partially reversed by CC (Fig. 3E). Taken together, apigenin induced ferroptosis in ALL cells, and that induction effect could be partially abolished by inhibition of AMPK signaling.

### Apigenin exerted an anti-leukemia activity by activating AMPK signaling

**Figure 4:**
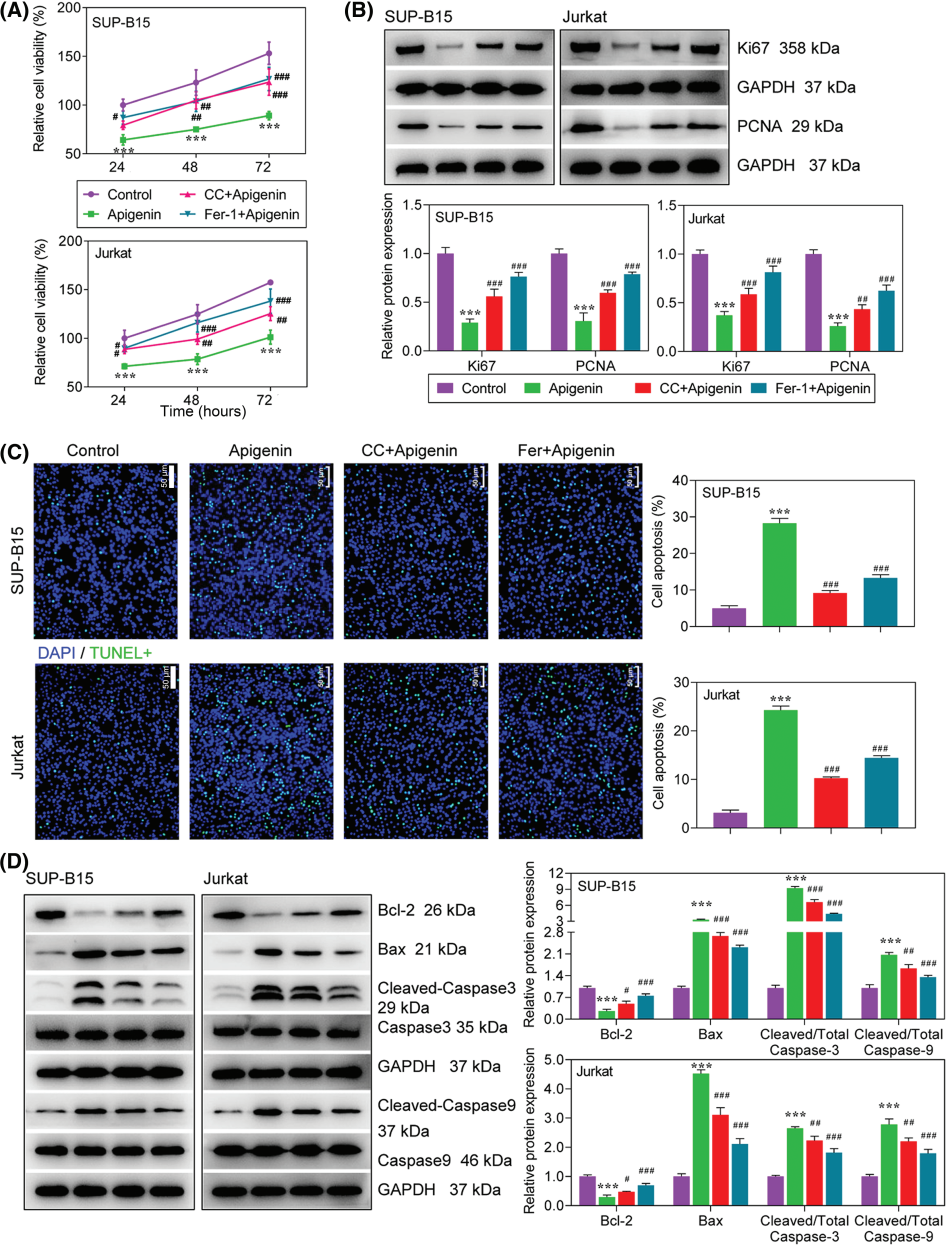
Apigenin exerted its anti-leukemia activity via activation of AMPK signaling. Cells were treated with compound C (10 μM), Fer-1 (0.1 μM) or apigenin (40 μM). (A) Cell viability was detected using the MTT assay. (B) The levels of Ki67 and PCNA expression at 24 h after treatment were determined by western blotting. (C) TUNEL assays at 24 h after treatment were conducted to evaluate cell apoptosis. (D) The expression of apoptosis-related proteins at 24 h after treatment was detected by western blotting. ***p < 0.001 *vs*. Control; ^#^*p* < 0.05, ^##^*p* < 0.01, ^###^*p* < 0.001 *vs*. apigenin.

To explore the role played by AMPK-mediated ferroptosis in the anti-leukemia activity of apigenin in ALL cells, SUP-B15 and Jurkat cells were pre-treated with a ferroptosis inhibitor (Fer-1) or an AMPK inhibitor (compound C), followed by treatment with apigenin. Next, the levels of cell proliferation and apoptosis were detected in the treated cells. Our data showed that the decrease in cell viability that occurred after apigenin treatment could be partially reversed by CC or Fer-1 treatment (Fig. 4A). As expected, the decreases in Ki67 and PCNA protein expression seen after apigenin treatment were partially abolished by CC or Fer-1 in both SUP-B15 and Jurkat cells (Fig. 4B). A cell apoptosis analysis showed that the additional treatment with CC or Fer-1 attenuated the apigenin-induced increase in apoptotic cells and also the changes in apoptosis-related protein expression (Figs. 4C and 4D), indicating that the pro-apoptotic activity of apigenin in ALL cells was partially achieved by activation of AMPK signaling.

## Discussion

**Figure 5:**
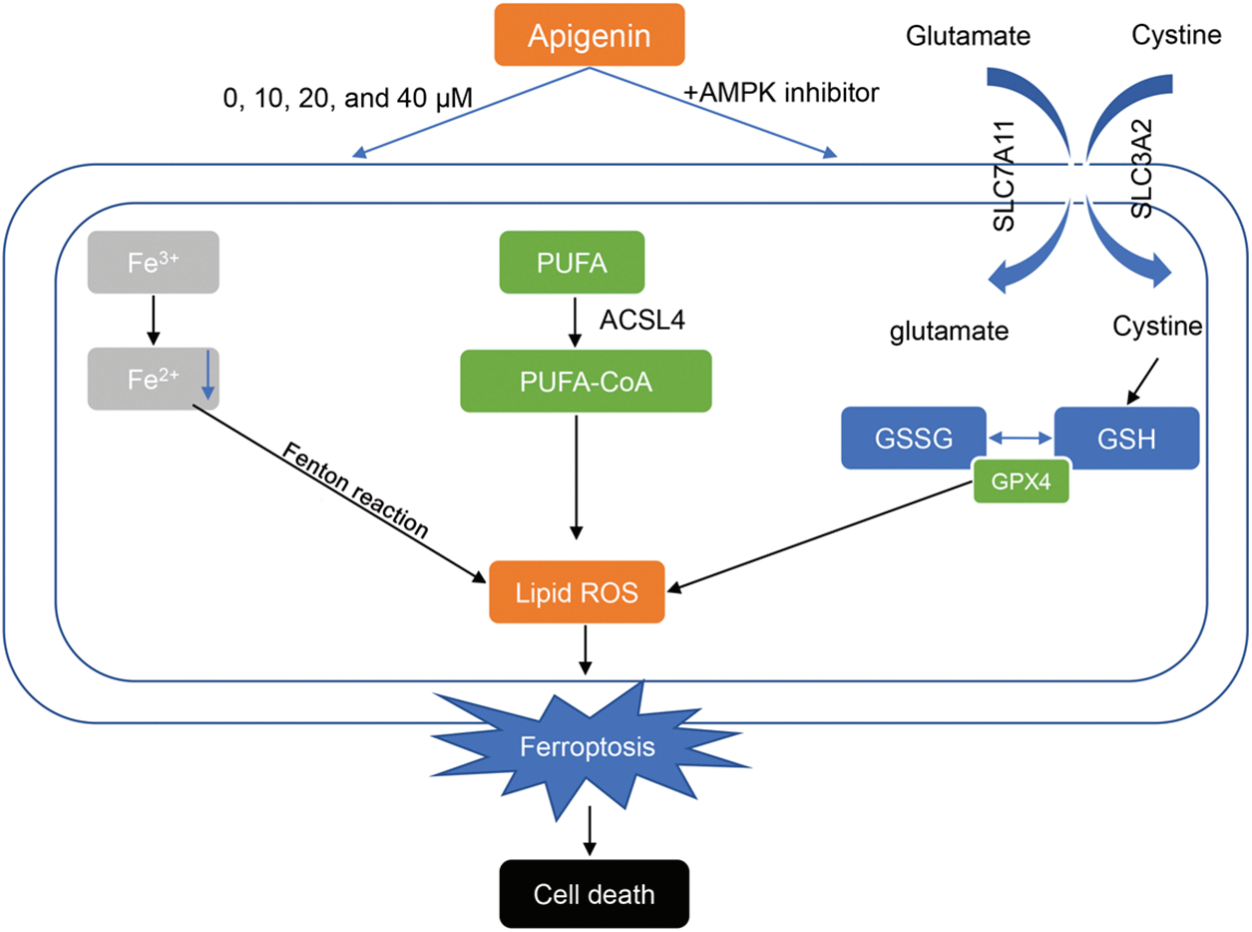
Apigenin facilitates the apoptosis of acute lymphoblastic leukemia cells via AMP-activated protein kinase-mediated ferroptosis. These two abbreviations represent two forms of glutathione: oxidized glutathione (GSSG) and reduced glutathione (GSH). GPX4: Glutathione Peroxidase 4. Lipid ROS: Lipid Reactive Oxygen Species. PUFA: polyunsaturated fatty acid. PUFA-CoA: Polyunsaturated fatty acid CoA refers to a compound in which coenzyme A (CoA) binds to polyunsaturated fatty acids.

ALL is a complicated disease, and its pathological mechanism is not fully understood. Despite the great advances made in treating ALL, ALL patients who suffer relapses still have a poor prognosis. Thus, it is essential to develop novel strategies for treating ALL. This study suggests apigenin as a potential agent for treating ALL. Our data showed that apigenin could remarkably inhibit ALL cell proliferation and promote apoptosis, and thus effectively impede the development of ALL (Fig. 5).

Apoptosis is a well-characterized form of programmed cell death that is also relevant to the removal of unwanted or damaged cells. Hence, it is recognized as a pivotal mechanism for cell survival, as well as a critical target of tumor therapeutics [19]. Current evidence confirms that apoptosis is an extremely significant aspect in treating ALL relapse. Furthermore, the modulation of apoptosis in patients with ALL relapse is aberrantly inactive, as demonstrated by a reduced Bax/Bcl-2 ratio and a decrease in spontaneous caspase-3 processing [20]. We found that apigenin was able to alter the Bax/Bcl-2 ratio in favor of apoptosis. Moreover, studies have shown that apigenin can induce apoptosis in multiple types of malignant tumors [21–24]. Additionally, Rahmani et al. [25] reported that apigenin exhibited an anti-cancer activity that is vital for cancer treatment. We found that apigenin exerted an inhibitory effect on apoptosis in ALL cells, which was consistent with a previous study that focused on the fact that combined use of apigenin with etoposide or cyclophosphamide had a better effect on promoting cell apoptosis [26]. Ferroptosis is recognized as one form of regulated cell death, and is characterized by the iron-dependent accumulation of lipid hydroperoxides, which can be useful for anti-cancer therapy [27,28]. However, the characteristics of ferroptosis in ALL are unclear. In recent years, increasing efforts have been made to investigate the role of ferroptosis in ALL. For example, Chen et al. [29] demonstrated that ALL progression and chemotherapy resistance could be predicated by a ferroptosis score, which could also serve as a prognostic indicator. It is reported that typhaneoside can induce ferroptosis and suppress cell proliferation in acute myeloid leukemia [30]. Similar results were reported by Birsen et al. [31], who found that ferroptosis was induced by a p53-targeted agent (APR246) in acute myeloid leukemia. In addition, the genetic inactivation of SLC7A11 or GPX4, which are anti-ferroptosis proteins, had a synergistic effect with APR-246 on promoting cell death. These findings suggest that ferroptosis is involved in the suppression of leukemia. Accordingly, our data revealed that apigenin caused a large induction of ferroptosis in ALL cells, as evidenced by increased levels of peroxidized lipids and Fe^2+^, as well as reduced levels of SLC7A11 or GPX4, suggesting that ferroptosis might be relevant to the anti-leukemia activity of apigenin.

AMPK works as a sensor of cellular energy status and a regulator of metabolism that is involved in regulating cell cycle progression [32,33]. Activated AMPK signaling has been confirmed to exert pro-apoptotic effects on leukemia cells [34]. For example, phenformin was found to halt the development of ALL via cell-autonomous AMPK activation [35]. Metformin induces ALL cell apoptosis effectively, and metformin-induced apoptosis was shown to be AMPK-dependent [36]. Here, we found an activation of AMPK following apigenin treatment. Furthermore, to determine whether the anti-leukemia activity of apigenin is AMPK-dependent, an AMPK inhibitor (CC) was used in several experiments. The results showed that CC not only attenuated apigenin-provoked ferroptosis, but also decreased the inhibitory effect of apigenin on cell progression in both SUP-B15 and Jurkat cells, demonstrating that inhibition of AMPK could partially hinder the anti-leukemia activity of apigenin, and AMPK is an important and indispensable pathway involved in the therapeutic effects of apigenin on ALL. Researchers have reported that an activated AMPK signaling pathway plays an important role in oxidative stress and ferroptosis by regulating NFE2-like bZIP transcription factor 2 (Nrf2) [37,38]. However, AMPK signaling might play a dual role in regulating ferroptosis in different cells or environments. Lee et al. [39] found that activated AMPK inhibits ferroptosis, while Zhao et al. [40] reported that activation of AMPK enhances ferroptosis. With regard to our current study, further experiments are needed to determine the mechanism by which ferroptosis can be regulated after apigenin-induced activation of AMPK.

## Conclusion

Collectively, our study provides novel insights into the regulatory role of apigenin in ALL. Apigenin hinders cell proliferation and promotes cell apoptosis and ferroptosis in ALL cells. Mechanistically speaking, the anti-leukemia activity of apigenin in ALL was partly achieved by activation of AMPK signaling. Therefore, apigenin might be considered as a promising therapeutic agent for the treatment of ALL. Although we have reported some meaningful conclusions, the detailed molecular mechanism by which apigenin influences ferroptosis and AMPK signaling remains to be revealed, and verification studies of the mechanism also need to be conducted. These types of studies will be the focus of our future work. Moreover, *in vitro* and *in vivo* transfection and verification experiments need to be performed to determine whether ferroptosis works through the AMPK signal pathway.

## Data Availability

The data that support the findings of this study are available from the corresponding author upon reasonable request.
